# Activated platelet-derived exosomal LRG1 promotes multiple myeloma cell growth

**DOI:** 10.1038/s41389-024-00522-5

**Published:** 2024-06-13

**Authors:** Meng Gao, Hang Dong, Siyi Jiang, Fangping Chen, Yunfeng Fu, Yanwei Luo

**Affiliations:** 1 Department of Blood Transfusion, The Third Xiangya Hospital, Central South University, Changsha, China; 2 Department of Hematology, The Third Xiangya Hospital, Central South University, Changsha, China

**Keywords:** Cancer microenvironment, Cell growth

## Abstract

The hypercoagulable state is a hallmark for patients with multiple myeloma (MM) and is associated with disease progression. Activated platelets secrete exosomes and promote solid tumor growth. However, the role of platelet-derived exosomes in MM is not fully clear. We aim to study the underlying mechanism of how platelet-derived exosomes promote MM cell growth. Flow cytometry, Western blot, proteome analysis, co-immunoprecipitation, immunofluorescence staining, and NOD/SCID mouse subcutaneous transplantation model were performed to investigate the role of exosomal LRG1 on multiple myeloma cell growth. Peripheral blood platelets in MM patients were in a highly activated state, and platelet-rich plasma from MM patients significantly promoted cell proliferation and decreased apoptotic cells in U266 and RPMI8226 cells. Leucine-rich-alpha-2-glycoprotein 1 (LRG1) was significantly enriched in MM platelet-derived exosomes. Blocking LRG1 in recipient cells using LRG1 antibody could significantly eliminate the proliferation-promoting effect of platelet-derived exosomes on MM cells. And high exosomal LRG1 was associated with poor prognosis of patients with MM. Mechanistic studies revealed that LRG1 interacted with Olfactomedin 4 (OLFM4) to accelerate MM progression by activating the epithelial-to-mesenchymal transition (EMT) signaling pathway and promoting angiogenesis. Our results revealed that blocking LRG1 is a promising therapeutic strategy for the treatment of MM.

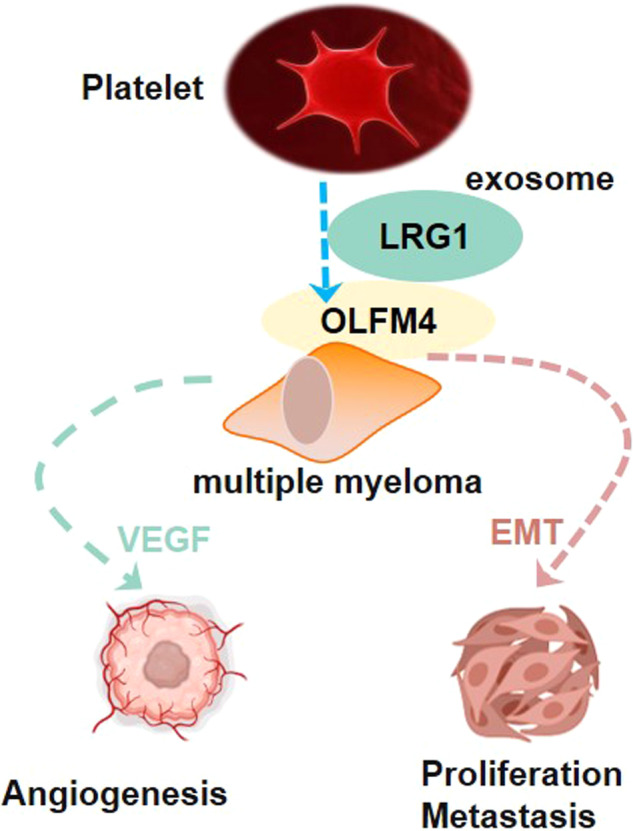

## Introduction

Multiple myeloma (MM), a common hematological malignancy, is characterized by the accumulation of plasma cells in the bone marrow and often induces anemia, osteolytic lesions, renal insufficiency, hypercalcemia, and immunodeficiency [1]. New treatments, such as immunomodulatory drugs, proteasome inhibitors, and autologous hematopoietic stem cell transplantation, have significantly increased the life expectancy of MM patients over the past few decades [2]. However, there is currently no cure for MM.

Studies have revealed that a hypercoagulable state is a hallmark for patients with multiple myeloma (MM) and is associated with disease progression [3]. Platelets are the major cellular component of blood, and studies have described their interaction with circulating tumor cells to support their proliferation, differentiation, and migration, in turn the tumor cells influence the coagulation function by producing many cytokines [4, 5].

On the other side, platelets promote primary tumor growth through angiogenesis, and support to evade the immune system [6]. Thus, high platelet activation has been associated with poor prognosis in patients with various tumors. In addition, studies have shown that platelet-derived exosomes can promote angiogenesis, extracellular matrix degradation, and release of adhesion and growth factors in various solid tumors, leading to tumor development and metastasis [7]. Platelet-derived exosomes contain proteins, genes, and non-coding RNAs, which are small and have good information transmission effects in the information transmission between platelets and tumor cells [8, 9]. Many studies have shown that proteins carried by exosomes undergo significant changes during the development of diseases, which can be used as ideal markers for disease diagnosis [10]. And exosomes separated from body fluids for monitoring significantly reduces the complexity of the sample and facilitates the detection of biomarkers. Therefore, exosomes provide a new idea and direction for disease diagnosis and marker exploration [11]. However, the underlying mechanism of how platelet-derived exosomes promote MM cell growth remains unclear.

In this study, we found that platelet-derived exosomes enriched LRG1 to promote the proliferation of MM cells. Mechanistic studies revealed that LRG1 interacted with OLFM4 to accelerate MM progression by activating the EMT signaling pathway and promoting angiogenesis. This study provides new insight that blocking LRG1 is a promising therapeutic strategy for the treatment of MM.

## Materials and methods

### Blood samples

A total of 80 (52 males and 28 females; age 42 to 75 years old) newly diagnosed MM patients without co-morbidities, including other cancers, diabetes, cardiovascular disease, and neurological disease in our hospital from October 2017 to April 2019 were enrolled. MM diagnosis, staging, and risk status were in accordance with the National Comprehensive Cancer Network Guidelines (3rd Edition, 2017). A total of 65 healthy subjects (43 males and 22 females; age, 40 to 78 years) were enrolled as the control group during the same period. The procedures in this study followed were in accordance with the ethical standards of the responsible committee on human experimentation and with the Helsinki Declaration of 1975, as revised in 2008, and was approved by The Third Xiangya Hospital ethics committee (NO.2017S124). All human participants have given written informed consent.

### Cell culture and treatment

U266, RPMI8226 cells were purchased from the cell bank of Shanghai Institute of Biology (Shanghai, China), and were cultured in 1640 complete medium with 10% extracellular vesicle-depleted Fetal Bovine Serum (cat no. A2720801, Gibco, USA) at 37 °C, 5% CO_2_ incubator. For LRG1-blocking treatment, the cells were treated with 5 μg/ml of the anti-LRG1 antibody C4 (Santa Cruz Biotechnology, Inc. Dallas, Texas, USA) for 48 h and used for further analysis. For exosome treatment, the cells were treated with exosomes collected from 10 ml peripheral blood for 48 h and used for further analysis.

### Platelet activation assay

Fasting venous blood was collected and anticoagulated with sodium citrate. Specimens were tested within 15 min after collection. 1 ml of whole blood samples were added with 1 μg human CD61-PERCP (Becton Dickinson Biosciences, cat: 340506), 1 μg CD62P PE (Becton Dickinson Biosciences, cat: 348107) for 15 min incubation. Then, the samples were fixed with ice-cold 1% paraformaldehyde and analyzed by Flow cytometry (FACSCalliber, BD Biosciences, San Diego, CA) for CD61, CD62P, and percent double positive.

### Preparation of platelet-rich plasma (PRP)

About 10 ml peripheral blood was drawn from MM patients and healthy controls into 8% sodium citrate anticoagulation tubes. Whole peripheral blood was centrifuged at low-speed centrifugation of 200 × *g* for 10 min to obtain a PRP. PRP were centrifuged at 2000 × *g* for 10 min to obtain platelet-poor plasma (PPP) from supernatants, and platelets were purified by resuspension in modified calcium-free Tyrode buffer.

### Determination of platelet aggregation rate

The platelet aggregation rate was measured in PRP by stimulating with ADP (10 μM, Sigma-Aldrich, St. Louis, MO, USA) and detected by turbidimetric method with aggregator (Beijing Pulisheng LBYNJ4, Beijing, China). The maximum platelet aggregation rate was recorded.

### Detection of proliferation by 5-ethynyl-2’- deoxyuridine (EdU) fluorescent staining

RPMI8226 and U266 cells were treated with platelet-rich plasma or exosomes for 48 h. The EdU solution (50 μmol/L) was added and incubated with cells for 2 h, and the nuclear were stained with DAPI (2 μg/ml) for 5 min.

### Detection of apoptosis by flow cytometry

After treatment with platelet-rich plasma or exosomes for 48 h, the RPMI8226 and U266 cells were obtained by centrifugation at 2000 × *g* for 5 min. A Annexin V-PE apoptosis detection kit (KeyGen Bio Tech, Nanjing, China) was used to measure the apoptotic cells according to the instructions. The results were analyzed by using BD FlowJo software (V10). The apoptotic cells included annexin V+ single positive cells plus annexin V+ PI+ positive cells.

### Western blot

The tumor tissue or the treated cells were collected to extract proteins by RIPA Lysis Buffer (Beyotime, Shanghai, China). The proteins (30 μg) were separated with SDS gel and transferred to the PVDF membrane. After blocked with 5% BSA for 1 h, the membranes were incubated with the primary antibodies (Bcl2 (Rabbit monoclonal [E17], dilution, 1:1000), Bax (Rabbit monoclonal [E63], dilution, 1:1000), GAPDH (Rabbit monoclonal [EPR16891], dilution, 1:1000), LRG1(Rabbit monoclonal [EPR12362], dilution, 1:1000), CD63 (Rabbit monoclonal [EPR5702], dilution, 1:1000), E-cadherin (Rabbit monoclonal [EP700Y], dilution, 1:1000), N-cadherin (Rabbit monoclonal [EPR22397-264], dilution, 1:1000), Vimentin (Rabbit polyclonal (V9), dilution, 1:1000), VEGF (Rabbit monoclonal [EPR9699], dilution, 1:1000), MPO (Rabbit monoclonal [EPR20257], dilution, 1:1000), SAA1 (Rabbit monoclonal [D9H4L41], dilution, 1:1000), Transferrin (Rabbit monoclonal [EPR18240-29], dilution, 1:1000); from Abcam, UK. S100A8 (Rabbit monoclonal [E4F8V], dilution, 1:1000), S100A9 (Rabbit monoclonal [D3U8M], dilution, 1:1000), PCNA ((Rabbit monoclonal [13110], dilution, 1:1000) from Cell Signaling Technology, USA) overnight at 4 °C. After washed with TBST, the membranes were incubated with secondary antibodies (Goat Anti-Rabbit IgG H&L (HRP), dilution, 1:5000, cat no. ab205718, Abcam) for 1 h. The bands were obtained by ChemiDoc™ XRS system (Bio-Rad). ImageJ was used to quantify the bands.

### Proteome analysis

The peripheral blood was obtained from three pairs of MM patients and healthy subjects matched by sex and age (Supplementary Table 1). Unlabeled quantitative protein spectrum was used to detect the expression of proteins in the samples. Protein identification was performed using Precursor Qvalue. Product ion peak area was used for protein quantification, and at least three product ions were selected for quantification; Students *t*-test was used for protein difference analysis; protein function analysis was based on GO, KEGG.

### Extraction and identification of PRP exosomes

The PRP was obtained as abovementioned, and centrifuged at 150,000×*g* for 120 min. The supernatant was discarded, and then 100 μL of PBS was added to resuspend the exosome pellet. The exosomes were identified by transmission electron microscope and analyzed by Nanoparticle tracking analysis (NTA, NanoSight NS300, Malvern, Cambridge, United Kingdom). The expressions of exosome-related marker proteins CD63 and specific platelet marker CD41 were detected by flow cytometry.

### Measurement of exosomal LRG1 levels

Exosomal LRG1 levels were quantified by Human LRG1 ELISA Kit (cat no. ab260066, Abcam, UK) according to the manufacturer’s protocol.

### Fluorescently labeled exosomes

The cell membrane was stained with wheat germ agglutinin (cat no. W11261, Thermo Fisher, USA) according to the manufacturer’s protocol. PKH26 dye (2 μM, Solarbio, Beijing, China) was added and incubated with the platelet-derived exosome suspension for 20 min at room temperature, protecting it from light. PKH26-labeled exosomes were co-incubated with U266 and RPMI8226 cells for 24 h. The cells were washed twice with PBS and then resuspended in a culture medium; The exosome tracking was observed by confocal microscopy.

### Plasmids construction and transfection

The full-length LRG1, OLFM1, OLFM2, OLFM3, and OLFM4, and their truncated fragments (LRG1Δ1: 13–310 aa; LRG1Δ2: 1–12, 264–310 aa; LRG1Δ3: 1–263 aa; OLFM4Δ1: 155-507 aa; OLFM4Δ2: 31–71, 245–507 aa; OLFM4Δ3: 31–234 aa) were directly synthesized by Songon Biotech Corporation (Shanghai, China), and were inserted into pCMV-N-HA (cat: D2733-1, Beyotime) or pCMV-N-Flag (cat: D2722-1, Beyotime, Shanghai, China) plasmids to construct the recombinant plasmids. The nucleotide sequences of all recombinant plasmids were verified by DNA sequencing (Songon Biotech Corporation, Shanghai, China)). For transfection, 20 nM plasmids or empty plasmids (control) that were mixed with Lip3000 were added into the U266 cells and incubated for 48 h. The transfected U266 cells were used for further analysis.

### Immunoprecipitation (IP)

Forty-eight hours after transfection of the expression plasmid, cell lysis buffer (containing protease inhibitors (10 μl, cat no. 78425, Thermo Fisher, USA) was added to lyse U266 cells for 30 min, and the supernatant was collected after centrifugation at 12,000 × *g* for 30 min. Anti-HA antibody (10 μg, Rabbit polyclonal, cat no. ab9110, Abcam) or Anti-Flag antibody (10 μg, Rabbit polyclonal, cat no. ab205606, Abcam) and 50 μl protein A/G-beads (B23202, Protein A/G immunoprecipitation magnetic beads, Bimake, USA) were added to the lysate (1 mg) respectively and incubated overnight at 4 °C with slow shaking. The protein A/G-beads were collected and was washed four times with 0.5 ml lysis buffer and centrifuged at 3000 × *g* for 2 min. The proteins were collected to perform a Western blot.

### Immunofluorescence staining

Forty-eight hours after transfection of each recombinant plasmid into cells, the cells were crawled, fixed with 4% paraformaldehyde, permeabilized with 0.1% triton, blocked with donkey serum for 1.5 h, and incubated with primary antibodies (E-cadherin (Rabbit monoclonal [EP700Y], dilution, 1:1000), N-cadherin (Rabbit monoclonal [EPR22397-264], dilution, 1:1000), Vimentin (Rabbit polyclonal, dilution, 1:1000), VEGF (Rabbit monoclonal [EPR9699], dilution, 1:1000); from Abcam, UK) overnight at 4 °C. PBS was used as negative control. Fluorescent secondary antibodies (AlexaFluor488 donkey anti-rabbit, cat no. A21206, green; Alexa Fluor 594 goat anti-rabbit, cat no. R37117, red; Thermo Fisher, USA) were incubated for 1 h at room temperature. DAPI were used to stain the nuclei. The images were captured under a fluorescence microscope (Leica, USA).

### Endothelial cell tube formation assay

The Matrigel matrix (growth factor reduced) and pipette were pre-cooled overnight at 4 °C. About 60 μl of Matrigel matrix was injected into a 96-well plate and refrigerated again at 4 °C overnight. The plated 96-well plate was incubated at 37 °C for 30 min, the HUVECs cells were resuspended in medium, and 100 μl of HUVECs cells (3 × 10^5^ cells/ml) was added to each well. About 100 μl of treated U266 and RPMI8226 culture supernatants were added to each well. The tube formation of HUVECs in each group was observed under an inverted microscope (Mateo TL, Leica, USA). ImageJ plugin Angiogenesis Analyzer was used to quantify the vessel formation, including branch points and capillary length.

### NOD/SCID mouse subcutaneous transplantation model

NOD/SCID mice (4–5-week-old male, 18–20 g, *n* = 5/group) were purchased from SJA Laboratory Animal Co. LTD (Changsha, China). 1.0 × 10^6^ U266 cells (100 μl) transfected with LRG1 or OLFM siRNA were subcutaneously injected into the mice. The tumor volume was measured every 2 days by investigators who was blinded to the group allocation during the experiment. The animal experiments were approved by the animal ethics committee of Central South University and strictly followed the institutional and national guide for the care and use of laboratory animals.

### Immunohistochemistry

After tumor tissue was taken, the tissues were routinely fixed, embedded, and serially sectioned with a thickness of 4 μm and dewaxed with xylene. Gradient ethanol hydration treatment, blocking endogenous peroxidase with 3% hydrogen peroxide solution, washing with PBS buffer (pH = 7.2–7.4), and antigen retrieval with EDTA (pH = 8.0), and then incubated with primary antibody (mouse anti-human Ki67, 1:500) in a 37-degree water bath for 1 h, and add EnVision secondary antibody in a water bath for 30 min, rinse with PBS buffer, and stained with freshly prepared DAB solution. The reaction time was controlled under the microscope, the color development was stopped with tap water, the nuclei were counterstained with Mayer, and observed under the microscope.

### TUNEL staining

The tumor tissue samples were embedded with paraffin and cut to a thickness of 4 μm. The cut sections were heated in an oven at 45 °C overnight. After deparaffinization and antigen retrieval, proteinase K was diluted to a solution of 20 μg/mL with 10 mmol/L Tris-hydrochloric acid (pH 7.4–7.8), and was used to treat the sample for 20 min at 37 °C. After being washed twice with phosphate buffer solution (PBS) for 5 min each, the samples were incubated with a TUNEL reaction mixture for 60 min at 37 °C. Nuclei were then counterstained with DAPI. Apoptotic cells positive for TUNEL staining were observed under a microscope.

### Immunofluorescence analysis for CD34 and vascular endothelial growth factor (VEGF)

Immunofluorescence detection of CD34 and VEGF was performed on the excised tumor tissues. The slides were fixed in methanol at −20 °C for 5 min, placed in phosphate buffered solution containing 0.05% Tween-20 for 5 min, and then blocked with PBS containing 5% BSA for 1 h. After incubation with antibodies CD34 and VEGF (1:100, Abcam, UK) for 1 h, the slides were gently rinsed three times with PBST (3 min/time), and then incubated with secondary antibodies conjugated with AlexaFluor488 goat anti-mouse and AlexaFluor568 goat anti-rabbit IgG (IgG; H + L; 1:200, Invitrogen, USA) for 30 min. Images were captured using a confocal microscope (PerkinElmer, USA). ImageJ was used to quantify the density of fluorescence.

### Statistics

Kaplan–Meier analysis was performed to generate overall survival (OS) curves. Comparisons between two groups of data were analyzed using Student’s *t*-test, and multiple sets of data were analyzed with one-way ANOVA; data were presented as the means ± SEM using GraphPad Prism 8.01. *P* values less than 0.05 indicates statistical significance.

## Results

### Activated platelet promotes multiple myeloma cell proliferation

**Fig. 1:**
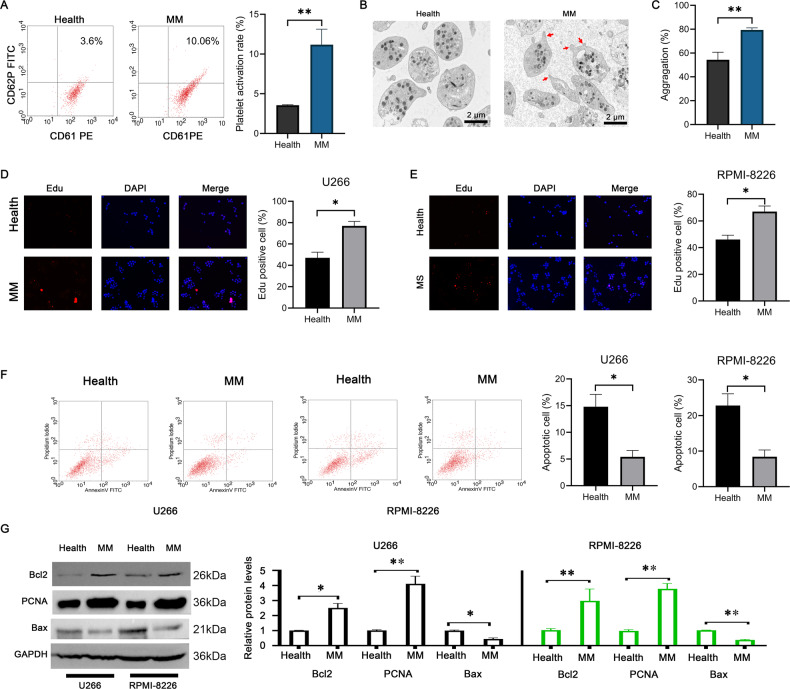
Platelets in MM patients are highly activated and promote MM cell proliferation. **A** The expression of platelet activation markers CD61 and CD62P was detected by flow cytometry. The platelet activation in MM patients is higher. **B** Scanning electron microscopy observed the morphology of platelets in the healthy control group and MM patients. The platelets in MM patients are spindle-shaped and protrude pseudopodia, and secrete microvesicles. **C** Platelet-rich plasma was stimulated with ADP to detect platelet aggregation rate. The platelet aggregation rate was significantly increased in MM patients. **D**, **E** MM cells U266 or RPMI8226 were treated with platelet-rich plasma derived from healthy people or MM patients, and cell proliferation was detected by Edu assay. Scale Bar, 200 μm. Platelet-rich plasma derived from MM patients significantly increased the number of Edu-positive cells in U266 and RPMI8226 cells. **F** Compared with the control group, platelet-rich plasma derived from MM patients significantly reduced the number of apoptotic cells in U266 and RPMI8226 cells. **G** MM patients-derived platelet-rich plasma significantly increased the expression of Bcl2 and decreased the expression of Bax compared with the control group. *P < 0.05, **P < 0.01.

Platelet activation is a common phenomenon in MM patients. To clarify the role of platelet activation in multiple myeloma, we collected peripheral blood from healthy people and MM patients and analyzed the expression of platelet surface proteins CD61 and CD62P by flow cytometry. We found that activated platelets in MM patients significantly increased (Fig. 1A), and scanning electron microscopy showed that platelets in multiple myeloma patients were significantly deformed and protruded pseudopodia (Fig. 1B). By isolating peripheral platelet-rich plasma and using adenosine diphosphate (ADP) to stimulate platelet activation, we found that patients with MM had higher platelet aggregation rates, suggesting that their platelets are more likely to be activated (Fig. 1C). We next tested the effect of platelet activation on the proliferation and apoptosis of multiple myeloma cells. Using platelet-rich plasma from healthy people and patients with MM to treat U266 and RPMI8226 cells, we found that multiple myeloma-derived platelet-rich plasma significantly increased the percentage of Edu-positive U266 and RPMI8226 cells (Fig. 1D, E), and decreased the percentage of apoptosis in U266 and RPMI8226 cells (Fig. 1F). Meanwhile, multiple myeloma-derived platelet-rich plasma significantly increased the expression of Bcl2, while inhibited the expression of Bax (Fig. 1G). These results suggest that patients with MM have higher levels of platelet activation and platelet activation can promote multiple myeloma cell proliferation.

### Platelet-derived exosomal LRG1 promotes proliferation of multiple myeloma cells

**Fig. 2:**
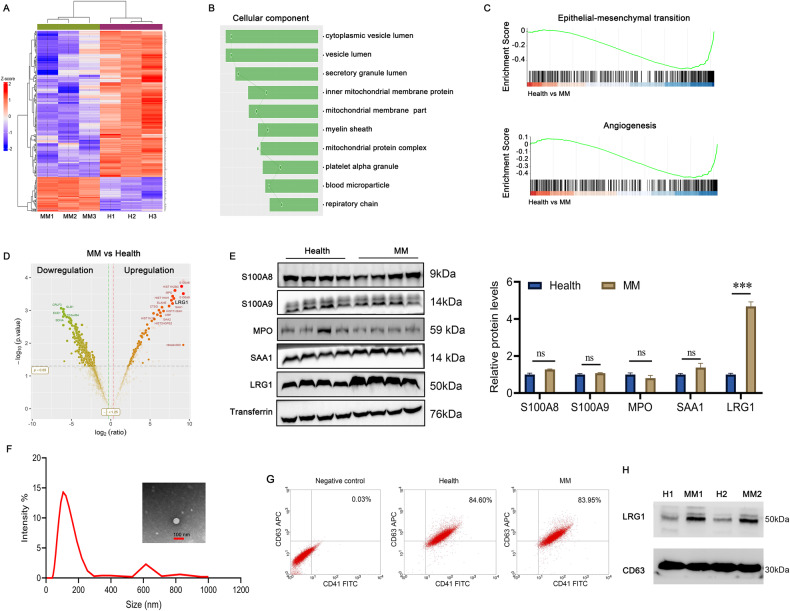
Proteomic analysis found that LRG1 enriched in platelet-derived exosomes. **A** Proteomic analysis of platelet-rich plasma derived from healthy individuals or MM patients, and a heat map showing the differential proteins. **B** GO analysis was performed on the differential proteins, and cell component analysis found that the differential proteins were closely related to vesicle secretion. **C** GSEA analysis of differential proteins found that differential proteins were closely related to EMT and angiogenesis. **D** Volcano plot showing significantly different proteins found in proteome analysis. **E** The expression of S100A8, S100A9, MPO, SAA1, and LRG1 in platelet-rich plasma derived from healthy people or patients with MM was verified by WB, and the expression of LRG1 was significantly increased in platelet-rich plasma derived from patients with MM. **F** The exosomes in platelet-rich plasma derived from MM patients were obtained, and the morphology and particle size of exosomes were analyzed by transmission electron microscopy and nanoparticle tracking analysis (NTA). **G** Exosomes expressing the specific platelet marker CD41 and exosome marker CD63 were sorted by flow cytometry. **H** The expression of LRG1 in platelet-derived exosomes was verified by WB. Compared with the control group, LRG1 expression was significantly increased in MM platelet-derived exosomes. ***P < 0.001.

Platelet-rich plasma contains a variety of biologically active proteins. To investigate functional proteins that promote the proliferation of multiple myeloma cells, we performed proteomic analysis of platelet-rich plasma. Compared with healthy people, there were 477 differentially expressed proteins in the platelet-rich plasma of patients with multiple myeloma. GO analysis found that these differential proteins were closely related to vesicle formation and blood microparticles. Gene Set Enrichment Analysis (GSEA) analysis found that these differential proteins were closely related to EMT and angiogenesis (Fig. 2A–D). We performed a western blot to verify the differential proteins, including S100A8, S100A9, MPO, SAA1, and LRG1. We further confirmed that LRG1, but not S100A8, S100A9, MPO, and SAA1, was significantly increased in platelet-rich plasma from patients with MM (Fig. 2E). We next focused on LRG1 for further investigation. Since these differential proteins in PRP were closely related to vesicle formation, we isolated exosomes from PRP (Fig. 2F), and sorted out platelet-derived exosomes (CD41^+^CD63^+^) by flow cytometry (Fig. 2G), and confirmed that LRG1 was significantly enriched in platelet-derived exosomes from patients with MM (Fig. 2H).

**Fig. 3:**
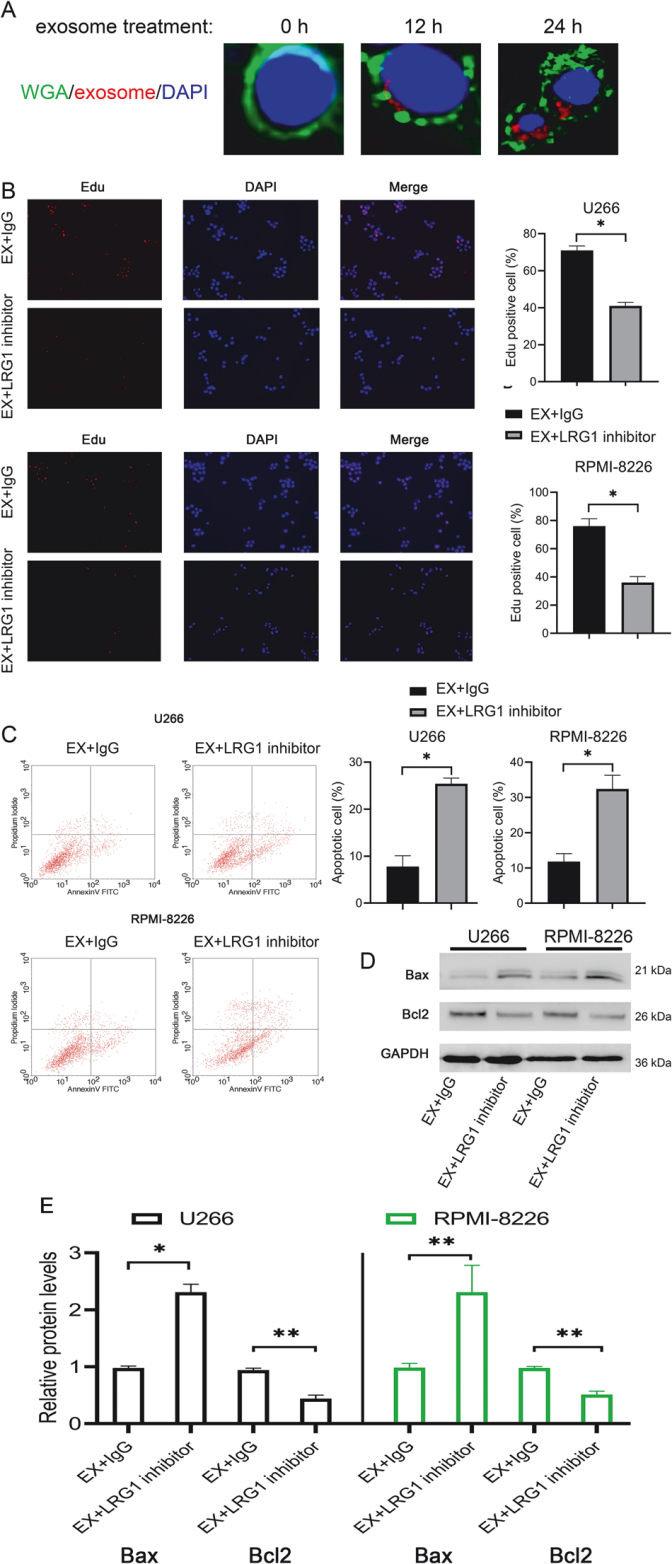
LRG1 inhibitor reversed the pro-proliferation effect of MM platelet-derived exosomes on U266 and RPMI8226 cells. **A** PKH26 was used to label exosomes, and wheat germ agglutinin (WGA, green) was used to label the membrane. The fusion of exosomes with U266 cells was observed under a fluorescence microscope. DAPI stained the nuclei. **B** Edu assay to detect cell proliferation. Compared with the MM platelet-derived exosome plus IgG group, the LRG1 inhibitor significantly reduced the number of Edu-positive cells in U266 and RPMI8226 cells. **C** Compared with the MM platelet-derived exosome plus IgG group, the LRG1 inhibitor significantly increased the number of apoptotic cells in U266 and RPMI8226. **D**, **E** LRG1 inhibitor significantly increased the expression of Bax and decreased the expression of Bcl2 compared with the MM platelet-derived exosome plus IgG group. *P < 0.05.

We treated U266 and RMPI8226 cells with platelet-derived exosomes from healthy control and MM patients, and found that MM-derived exosomes dramatically increased the percentage of Edu-positive U266 and RPMI8226 cells, and decreased the percentage of apoptosis in U266 and RPMI8226 cells compared with healthy control (Fig. S1). To investigate the function of platelet-derived exosomal LRG1 in multiple myeloma, we treated U266 and RPMI8226 cells with platelet-derived exosomes from MM patients and an LRG1-blocking monoclonal antibody (LRG1 inhibitor) [12–14]. By labeling exosomes with PKH26 and cell membrane with wheat germ agglutinin, tracer analysis found that exosomes had been uptake into tumor cells (Fig. 3A). The Edu-positive rate in U266 and RPMI8226 cells treated with the exosomes was 70%, while the Edu-positive rate was significantly reduced to 40% after treatment with LRG1 inhibitor (Fig. 3B). Likewise, LRG1 inhibitor treatment significantly upregulated the number of apoptotic MM cells (Fig. 3C), as well as inhibited Bcl2 and increased Bax expression (Fig. 3D, E). These results suggest that LRG1 is an important pro-proliferation protein in platelet-derived exosomes from patients with MM.

### LRG1 regulates EMT and angiogenesis in multiple myeloma cells via OLFM4

**Fig. 4:**
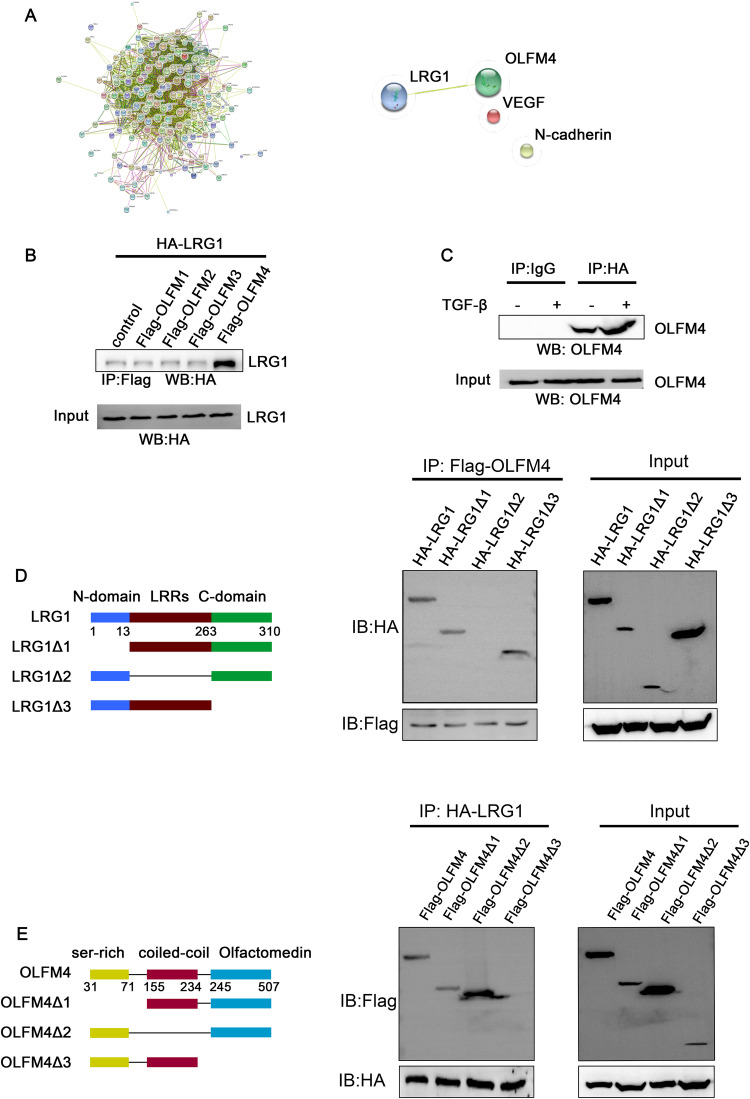
Interaction between LRG1 and OLFM4. **A**, **B** Proteomic analysis of possible interacting molecules of LRG1, of which OLFM4 is a predicted interacting molecule of LRG1 and is closely related to VEGF and N-cadherin. **C** Co-IP detection of interactions between LRG1 and OLFM family molecules. LRG1 can interact with OLFM4. **D** TGF-β enhances the interaction between LRG1 and OLFM4. **E** Different truncated LRG1 expression plasmids were constructed. Co-IP found that LRG1 without LRRs domain could not interact with OLFM4. **E** Different truncated OLFM4 expression plasmids were constructed. Co-IP found that OLFM4 without the Olfactomedin domain could not interact with LRG1.

Since PRP proteins are closely related to EMT and angiogenesis, we found that LRG1 interacts with OLFM4 and is closely related to VEGF and N-cadherin through protein interaction analysis (Fig. 4A). Through Co-IP experiments, we confirmed that LRG1 and OLFM4 could bind to each other, but did not interact with OLFM1, OLFM2, and OLFM3 (Fig. 4B). Studies have shown that TGF-β can enhance the function of LRG1. We treated U266 cells with TGF-β and performed Co-IP experiments to further confirm the interaction between LRG1 and OLFM4 (Fig. 4C). We further investigated the domains of LRG1 interacting with OLFM4. LRG1 contains one N-terminal domain, one C-terminal domain, and eight LRR domains. We constructed LRG1 full-length and various truncated plasmids (Fig. 4D). The results of Co-IP experiments showed that LRG1 could not bind to OLFM4 without the LRR domain (Fig. 4D), suggesting that LRG1 binds to OLFM4 through the LRR domain. On the other hand, OLFM4 contains a ser-rich domain at the N-terminal, an olfactomedin domain at the C-terminal, and a coiled-coil domain in the middle. We constructed OLFM4 full-length and various truncated plasmids (Fig. 4E). Co-IP experiments showed that OLFM4 could not bind to LRG1 without the olfactomedin domain (Fig. 4E), suggesting that OLFM4 binds to LRG1 through the olfactomedin domain.

**Fig. 5:**
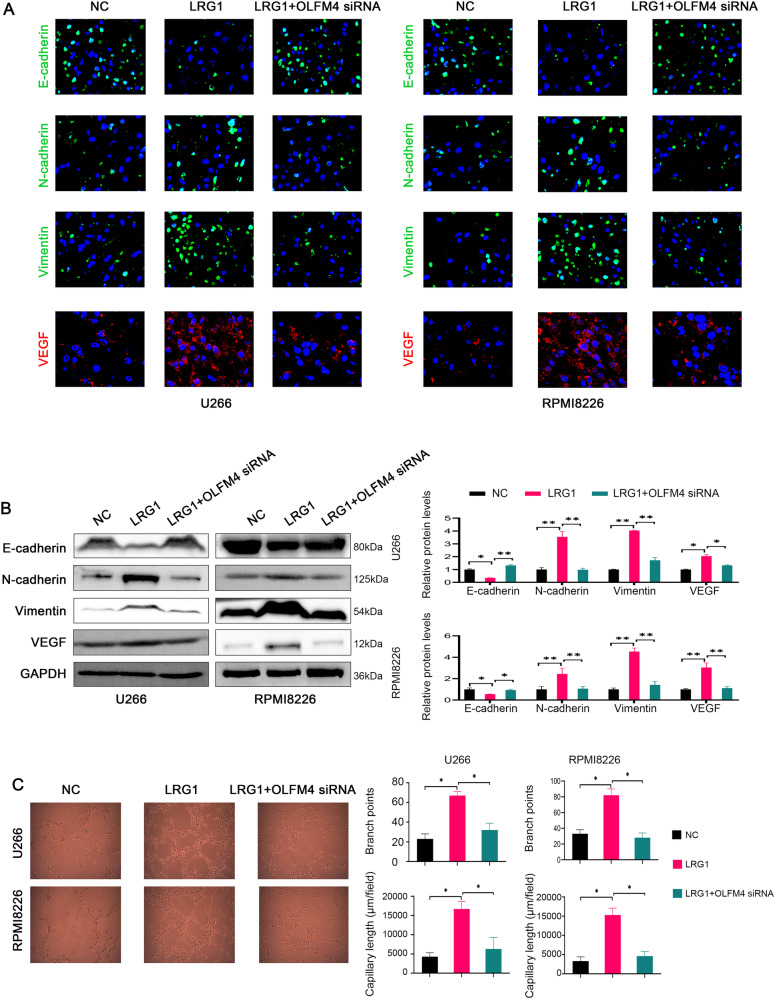
LRG1 promotes EMT and angiogenesis through OLFM4 in vitro. **A** Representative immunofluorescence images of E-cadherin, N-cadherin, Vimentin, VEGF. LRG1 significantly inhibited the expression of E-cadherin and increased the expression of N-cadherin, Vimentin, and VEGF, while OLFM4 siRNA transfection significantly reversed the effect of LRG1 on these molecules. **B** The expression of E-cadherin, N-cadherin, Vimentin, and VEGF was detected by WB. **C** In vitro blood vessel formation assay. LRG1 significantly increased branch points and capillary length in vascular endothelial cells in vitro, whereas OLFM4 siRNA transfection significantly attenuated the effect of LRG1 on angiogenesis. *P < 0.05, **P < 0.01.

It has been shown that LRG1 is involved in angiogenesis, and our results suggest that LRG1 is closely related to angiogenesis and EMT. As the markers of EMT signaling, upregulation of N-cadherin and vimentin and downregulation of E-cadherin indicate activation of EMT signaling. And VEGF is a pro-angiogenic factor for angiogenesis. We overexpressed LRG1 and knocked down OLFM4 in U266 and RPMI8226 cells (Fig. S2), and found that LRG1 overexpression significantly promoted the expression of N-cadherin and vimentin, but decreased the expression of E-cadherin, while upregulated VEGF level. However, interfering with OLFM4 reversed LRG1-mediated promotion of EMT and angiogenesis (Fig. 5A, B). We treated endothelial cells with isolated culture supernatant exosomes or with exosome-poor culture supernatant from U266 and RMPI8226 culture medium, and found that the more vessel formation was observed in exosomes-treated group than exosome-poor culture supernatant treated group, suggesting that exosomes contributed to vessel formation (Fig. S3). In addition, the exosomes from U266 and RPMI8226 cells overexpressing LRG1 stimulated endothelial cells to form a vessel-like morphology evaluated by increased branch points and capillary length, which was significantly reduced after interference with OLFM4 (Fig. 5C). These results suggest that exosomal LRG1 promotes EMT in multiple myeloma cells through OLFM4 and promotes angiogenesis.

### LRG1 promotes multiple myeloma growth and tumor angiogenesis in vivo

**Fig. 6:**
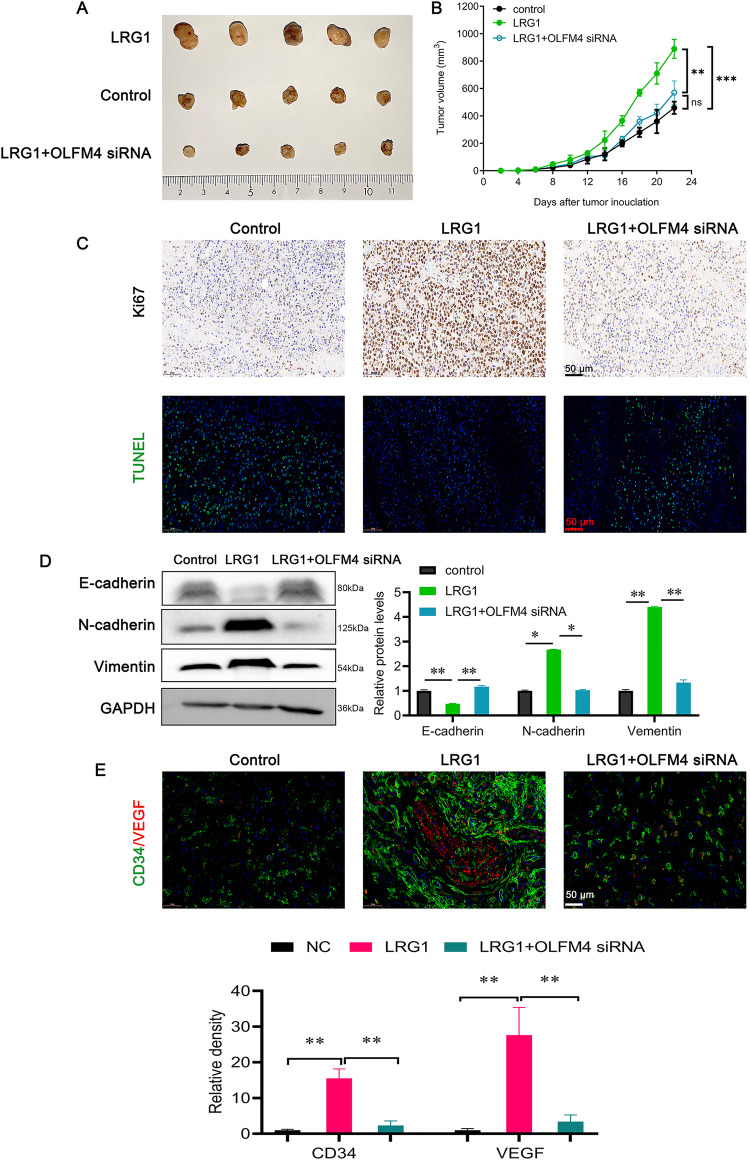
The LRG1/OLFM4 axis promotes tumor growth in vivo by enhancing EMT and angiogenesis. **A**, **B** Subcutaneous tumorigenesis experiments showed that LRG1 significantly promoted tumor growth, while OLFM4 interfering significantly inhibited tumor growth. **C** Representative immunohistochemical images of Ki67 and TUNEL staining. LRG1 significantly increased the expression of Ki67, while interfering with OLFM4 significantly decreased the expression of Ki67. LRG1 significantly reduced the number of TUNEL-positive cells, whereas interfering with OLFM4 significantly increased the number of TUNEL-positive cells. **D** The expression of E-cadherin, N-cadherin, and Vimentin was detected by WB. LRG1 significantly inhibited the expression of E-cadherin and increased the expression of N-cadherin and vimentin, while OLFM4 siRNA transfection significantly reversed the effect of LRG1 on these molecules. **E** Representative immunofluorescence images of CD34 and VEGF. LRG1 inhibition significantly increased VEGF expression and angiogenesis, while OLFM4 siRNA transfection significantly reduced VEGF expression and angiogenesis. *P < 0.05, **P < 0.01.

We next confirmed the function of LRG1 in vivo by subcutaneous tumorigenesis experiments of multiple myeloma cells. Overexpression of LRG1 significantly increased the subcutaneous tumor size, and interference with OLFM4 reversed the LRG1-mediated growth-promoting effect (Fig. 6A, B). By immunohistochemistry and TUNEL staining, we found that LRG1 overexpression enhanced the expression of the proliferation marker Ki67 and reduced the number of TUNEL-positive cells, while interference with OLFM4 reversed these effects mediated by LRG1 (Fig. 6C). Furthermore, LRG1 significantly promoted EMT behavior of multiple myeloma cells in vivo, whereas OLFM4 interference significantly attenuated EMT signaling (Fig. 6D). The results of immunofluorescence showed that the tumors in the LRG1-overexpressing group formed more vascular-like structures (CD34 positive, green) and secreted more VEGF (red), while OLFM4 knockdown significantly reduced the vascular-like structures and VEGF secretion in the tumors (Fig. 6E). Thus, LRG1 promotes multiple myeloma cell EMT and tumor angiogenesis via OLFM4 in vivo.

### Platelet exosome-derived LRG1 is a prognostic marker in patients with multiple myeloma

**Fig. 7:**
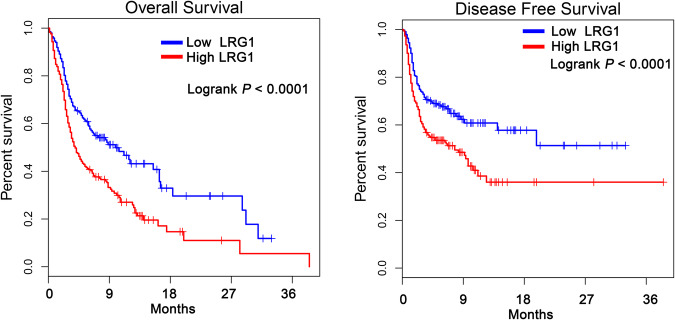
High LRG1 expression is a poor prognostic factor in MM patients. Survival curve analysis of the relationship between the expression of platelet-derived exosomal LRG1 and the overall survival and disease-free survival of MM patients. MM patients with high LRG1 expression had shorter overall survival and disease-free survival.

We next investigated the relationship between the expression of platelet-derived exosomal LRG1 and the prognosis of patients with MM. The peripheral blood samples from 80 patients with MM were collected and used to detect the expression of exosomal LRG1. The patients with multiple myeloma were divided into patients with low LRG1 expression (mean: 41. 6 μg/ml, maximum: 56.5 μg/ml, minimum: 15.4 μg/ml) and high LRG1 expression (mean: 73.6 μg/ml, maximum: 135.7 μg/ml, minimum: 57.2 μg/ml) according to the median of LRG1 expression. LRG1 expression was not significantly associated with age and gender in MM patients, but was significantly associated with Cytogenetic Risk, DSS, and ISS stage (Table 1). In addition, patients with high LRG1 expression had lower overall survival and disease-free survival than patients with low LRG1 expression (Fig. 7). These results suggest that platelet-derived exosomal LRG1 is a prognostic marker in patients with MM.

**Table 1:** Clinical association between LRG1 levels and clinicopathological variables of patients with multiple myeloma.

Variable	LRG1 levels		*χ*^2^ test *p* value
	Low expression (*n* = 40)	High expression (*n* = 40)	
**Age**			0.8213
<50	16	18	
≥50	24	22	
**Gender**			0.8149
Male	25	27	
Female	15	13	
**Cytogenetic Risk (%)**			0.0392*
High	11	21	
Standard	29	19	
DSS			0.0243*
I-II	27	16	
III	13	24	
ISS			0.0129*
I-II	24	12	
III	16	28	

*DSS* Durie-Salmon staging, *ISS* international staging system.

## Discussion

Studies have shown that platelets are involved in the occurrence and development of tumors, including tumor growth and metastasis, and immune escape, and platelets may have interactions with local and distant tumor cells through the secretion of exosomes [15, 16]. Tumor-secreted platelet factors have been shown to increase platelet aggregation, which in turn accelerates neoplastic tumorigenesis [17]. On the other hand, platelet-derived exosomes can stimulate cancer cells to express mitogen-activated protein kinases to promote proliferation and invasion [18]. Platelet-derived exosomes are significantly elevated in most malignant tumors [19]. For example, patients with colon cancer are often accompanied by changes in coagulation activity with high levels of platelet-derived exosomes in the systemic circulation and shortened clotting time that results in the blood of colon cancer patients being in the hypercoagulable state [20, 21]. In this study, we found that peripheral blood platelets in MM patients were in a highly activated state, and platelet-rich plasma from MM patients significantly promoted the proliferation of MM cells and inhibited apoptosis. Further analysis found that platelet-derived exosomes play an important role in enhancing MM proliferation.

Over 95% of circulating microparticles are derived from platelets, and these extracellular vesicles contain genetic material (microRNAs, messenger RNAs), proteins, and small molecules that can regulate the function of other cells [22]. After platelet activation, exosomes are released, and they carry proteins to receptor cells to play a regulatory role. Some studies have analyzed the differences in the exosomal protein composition of normal human bone marrow mesenchymal stem cells and bone marrow mesenchymal stem cells of patients with MM by proteomics, and found that IL-6, C-C Motif Chemokine Ligand 2 (CCL2) and fibronectin play a role in bone marrow mesenchymal stem cells of patients with multiple myeloma [23], and it’s upregulation in stromal cells were closely related to the characteristics of multiple myeloma. We here found that LRG1 was significantly enriched in MM platelet-derived exosomes. Blocking the effect of LRG1 in recipient cells by LRG1 antibody could significantly eliminate the proliferation-promoting effect of platelet-derived exosomes on MM cells. It is suggested that exosomal LRG1 is a key protein that promotes MM progression.

LRG1 (leucine-rich-alpha-2-glycoprotein 1), as one of the highly conserved members of the leucine-rich repeat (LRR) protein family, has been found that not only mediates protein–protein interactions, but also plays an important role in cell survival, apoptosis, and signal transduction [24, 25]. In recent years, studies have found that the upregulation of LRG1 protein is associated with the pathogenesis of various tissue malignancies, such as squamous cell lung cancer, colorectal cancer, ovarian cancer, and pancreatic cancer [26]. Recent studies have found that LRG1 can promote angiogenesis, especially the growth of abnormal blood vessels [27]. Abnormal angiogenesis promotes tumor growth and metastasis. The specificity of promoting abnormal blood vessel growth makes the LRG1 protein an important target for the treatment or intervention of abnormal blood vessel growth [28]. The LRG1 protein exerts its role in promoting abnormal angiogenesis by altering the signaling of a multifunctional secreted growth factor TGF-β [29]. It is a very promising future treatment to investigate the activity of blocking LRG1 for the intervention of angiogenesis-related diseases.

However, the current study of LRG1 protein is not comprehensive. For example, LRG1 promotes angiogenesis through TGF-β signaling, and TGF-β not only regulates the maintenance of normal healthy blood vessels, but also regulates the growth of unnecessary harmful blood vessels. How exactly TGF-β promotes the two opposite effects is still biologically controversial, and further experiments are needed to clarify. Through proteomic analysis, we found that LRG1 interacts with OLFM4 and is closely related to angiogenesis and EMT, and in vitro experiments confirmed that LRG1 binds to the Olfactomedin domain of OLFM4 through its LRR domain. OLFM4 is a member of the protein family containing the Olfactomedin domain and is an OLFM-related glycoprotein [30]. The expression of OLFM4 protein is increased in gastric cancer, colorectal cancer, cervical cancer, and other tissues, has an anti-apoptotic effect, and promotes tumor cell growth [31, 32]. Studies have shown that Lgr5 may be a potential marker of colorectal cancer stem cells, and OLFM4 is a marker of Lgr5 stem cells [33]. In the peripheral blood of colorectal cancer, those with high expression of OLFM4 are prone to distant tumor metastasis, and the prognosis is relatively poor. Those with high expression of OLFM4 in pancreatic cancer tissue have a poor prognosis [34]. Whether OLFM4, as a marker of LRG5 stem cells, is also a marker of origin cells of other human malignant tumors deserves further study. The increased expression of OLFM4 in cells may cause abnormal regulation of cell biological behaviors such as intercellular adhesion, cell cycle, and apoptosis, thereby leading to the development of tumors [35]. In multiple myeloma, plasma cells interact with the microenvironment, the continuous formation of bone marrow blood vessels, and the continuous promotion of tumor cell proliferation, evolution, and metastasis [36]. Neovascularization in multiple myeloma has become an important indicator of poor prognosis in myeloma [37]. Much research has been done on the function of platelets in various solid tumors, while the mechanism by which platelets regulate MM is not fully understood [38]. In this study, through downstream signaling pathway analysis, we found that LRG1 activated the EMT pathway and stimulated MM cells to release VEGF through OLFM4. Angiogenesis in tumors may be caused by a variety of cytokines and a large amount of vascular endothelial growth factor [39]. VEGF may be an autocrine factor of myeloma cells, and is an effective ligand for endothelial cells and a powerful stimulator of angiogenesis [40]. Persistent platelet activation-derived transforming growth factor beta promotes the process of epithelial-mesenchymal transition through the nuclear factor-κB pathway, which promotes tumor cell colonization in distant organs, while small platelet exosomes readily pass through various in vivo barriers, targeting colonization sites promote metastasis and invasion of cancer cells [41, 42]. Here, we further confirmed that the LRG1/OLFM4 axis promotes MM growth by activating EMT and pro-angiogenic pathways in vivo. There are some limitations of the present study, for example, it is not clear how exosomes enriched for LRG1, and whether exosomal LRG1 can be distantly delivered to promote tumor cell metastasis, its relationship with chemoresistance in MM is not clear, and whether it is associated with immunotherapy still needs further elucidation.

## Conclusion

We revealed a close interaction between platelets and tumor cells in MM patients. Activated platelets stimulate the proliferation of MM cells via exosomes. Mechanistic studies have found that exosomal LRG1 activates EMT and pro-angiogenic pathways by binding to OLFM4 to promote the growth of MM. Therefore, blocking the activity of LRG1 is a promising therapeutic option for the treatment of MM.

### Supplementary information

supplemental materials

## Data Availability

The datasets used and/or analysed during the current study are available from the corresponding author on reasonable request.
