# Supplementary material for: Activated platelet-derived exosomal LRG1 promotes multiple myeloma cell growth
Source: Oncogenesis. 2024 Jun 13;13(1):21. doi: 10.1038/s41389-024-00522-5 (PMC11176168; doi:10.1038/s41389-024-00522-5)
Supplement: Supplementary file 1 — supplemental materials [file 41389_2024_522_MOESM1_ESM.docx]

**Activated platelet-derived exosomal LRG1 promotes multiple myeloma cell growth**

**Supplementary Table 1 Clinicopathological variables of patients with multiple myeloma and healthy donor used in proteomics**

| **Subjects** | **Age** | **gender** | **Cytogenetic Risk** | **DSS** | **ISS** |
| --- | --- | --- | --- | --- | --- |
| MM patient 1 | 43 | male | high | II | I |
| MM patient 2 | 50 | male | high | II | II |
| MM patient 3 | 57 | male | high | III | III |
| Healthy control 1 | 40 | male | - | - | - |
| Healthy control 2 | 44 | male | - | - | - |
| Healthy control 3 | 53 | male | - | - | - |

DSS, Durie-Salmon staging; ISS, international staging system.


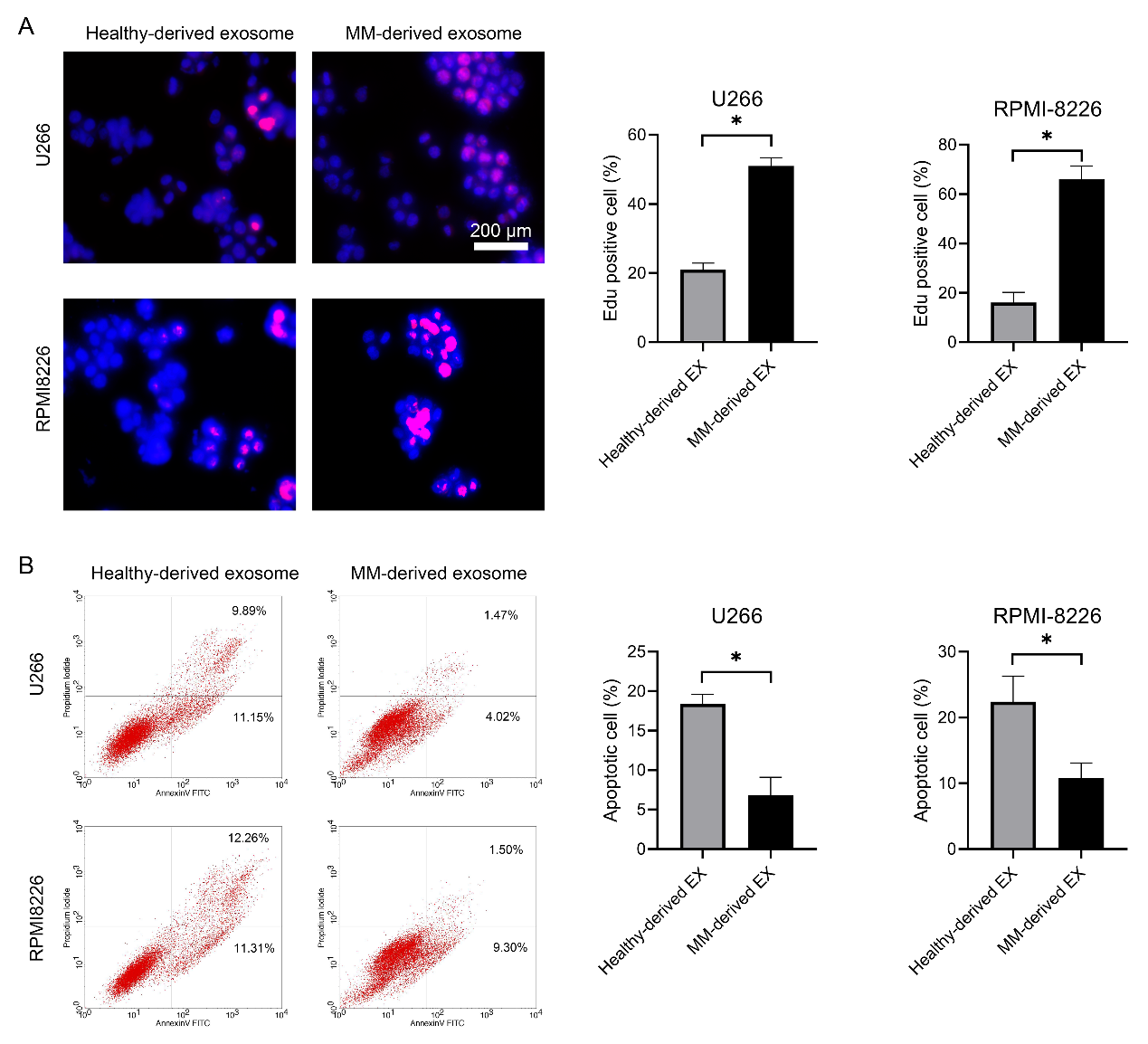


**Figure S1 The pro-proliferation effect of MM platelet-derived exosomes on U266 and RPMI8226 cells.** (A) Edu assay to detect cell proliferation. Compared with the healthy platelet-derived exosome group, MM platelet-derived exosome significantly increased the number of Edu-positive cells in U266 and RPMI8226 cells. (B) Compared with the healthy platelet-derived exosome group, MM platelet-derived exosome significantly reduced the number of apoptotic cells in U266 and RPMI8226. *P<0.05.


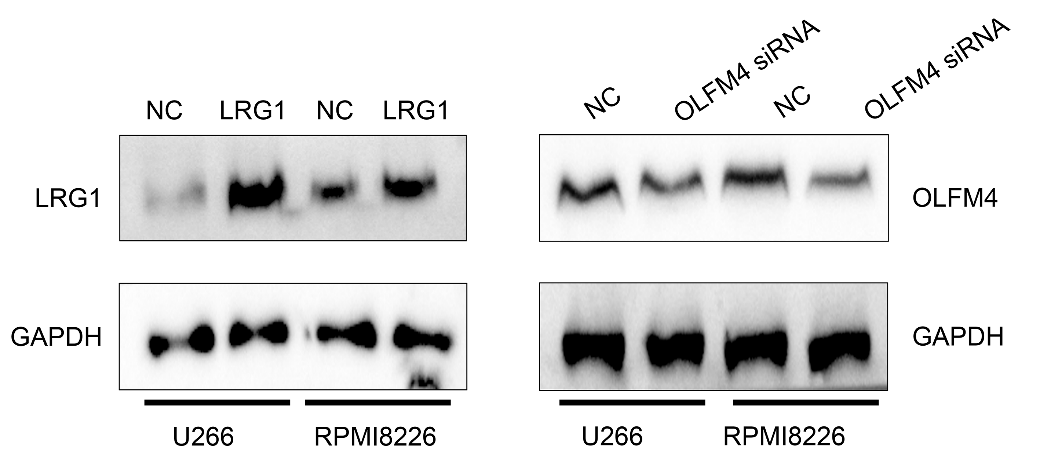


**Figure S2 LRG1 was overexpressed and OLFM4 was downregulated after transfection in U266 and RPMI8226 cells.** Western Blot was performed to detect the expression of LRG1 and OLFM4 after expressed plasmid or siRNA transfection in U266 and RPMI8226 cells.


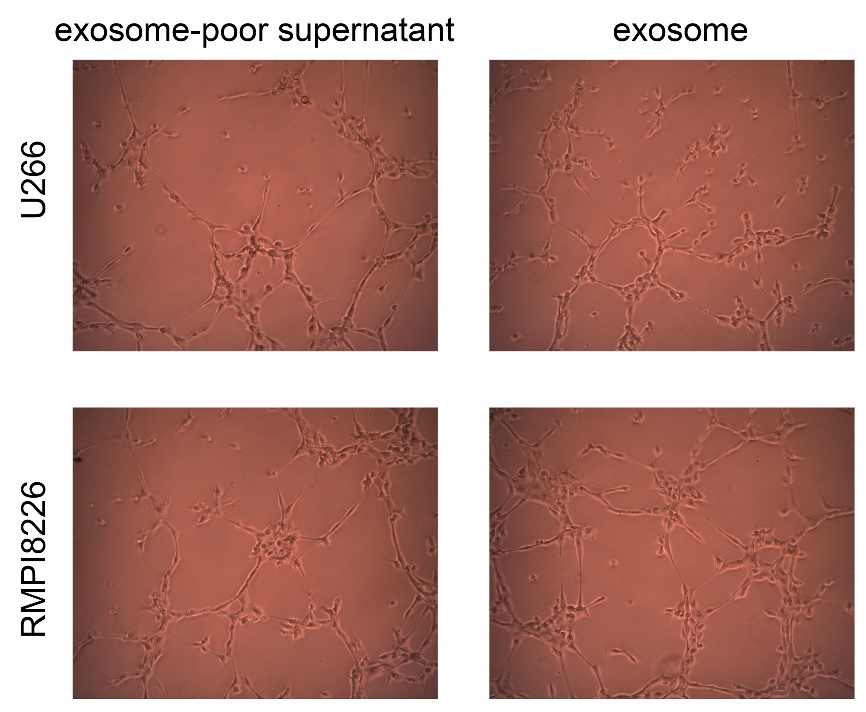


**Figure S3 Tumor cell derived exosomes contributed to vessel formation.** Exosomes were isolated from culture supernatant of U266 and RMPI8226 cells and used to treat HUVEC cells. The vessel formation was observed under microscopy. Exosome-poor culture supernatant was used as control.
